# A Systematic Review of Re-Identification Attacks on Health Data

**DOI:** 10.1371/journal.pone.0028071

**Published:** 2011-12-02

**Authors:** Khaled El Emam, Elizabeth Jonker, Luk Arbuckle, Bradley Malin

**Affiliations:** 1 Electronic Health Information Laboratory, CHEO Research Institute, Ottawa, Canada; 2 Department of Paediatrics, University of Ottawa, Ottawa, Canada; 3 Department of Biomedical Informatics, Vanderbilt University, Nashville, Tennessee, United States of America; 4 Department of Electrical Engineering and Computer Science, Vanderbilt University, Nashville, Tennessee, United States of America; Johns Hopkins Bloomberg School of Public Health, United States of America

## Abstract

**Background:**

Privacy legislation in most jurisdictions allows the disclosure of health data for secondary purposes without patient consent if it is de-identified. Some recent articles in the medical, legal, and computer science literature have argued that de-identification methods do not provide sufficient protection because they are easy to reverse. Should this be the case, it would have significant and important implications on how health information is disclosed, including: (a) potentially limiting its availability for secondary purposes such as research, and (b) resulting in more identifiable health information being disclosed. Our objectives in this systematic review were to: (a) characterize known re-identification attacks on health data and contrast that to re-identification attacks on other kinds of data, (b) compute the overall proportion of records that have been correctly re-identified in these attacks, and (c) assess whether these demonstrate weaknesses in current de-identification methods.

**Methods and Findings:**

Searches were conducted in IEEE Xplore, ACM Digital Library, and PubMed. After screening, fourteen eligible articles representing distinct attacks were identified. On average, approximately a quarter of the records were re-identified across all studies (0.26 with 95% CI 0.046–0.478) and 0.34 for attacks on health data (95% CI 0–0.744). There was considerable uncertainty around the proportions as evidenced by the wide confidence intervals, and the mean proportion of records re-identified was sensitive to unpublished studies. Two of fourteen attacks were performed with data that was de-identified using existing standards. Only one of these attacks was on health data, which resulted in a success rate of 0.00013.

**Conclusions:**

The current evidence shows a high re-identification rate but is dominated by small-scale studies on data that was not de-identified according to existing standards. This evidence is insufficient to draw conclusions about the efficacy of de-identification methods.

## Introduction

The availability of de-identified data has been critical for population health research, health services research, and public health. De-identification is the act of reducing the information content in data to decrease the probability of discovering an individual's identity. Over the past several decades, a number of different metrics and methods have been developed, and applied, to de-identify data [1]. De-identification has become a key component of various privacy statutes and regulations, especially in the context of health data [2], [3]. These provide strong incentives for its application when person-specific information is disclosed for secondary purposes (i.e., purposes beyond the initial reason for data collection). Additionally, in the context of health research, many institutional review boards will waive the consent requirement if the data is de-identified [4], [5].

Yet, there is a growing view that there has been a failure of de-identification [6], [7]. In particular, it has been claimed that data can be re-identified with relative ease, thus casting doubt on the ability to protect personal information from privacy invasions. This argument has been invoked to substantiate calls for legislative and regulatory changes in court cases [8] and in the peer-reviewed scientific literature [9], [10], [11], [12].

The importance of this claim cannot be overstated because there are significant policy implications at stake. Should there be a failure of de-identification, there would be at least three consequences on the practice of disclosing data for secondary purposes, such as health research [13]: (i) it may be necessary to obtain consent or authorization from patients before disclosure, (ii) there would be less incentive to de-identify data, and (iii) the likelihood of data breaches would increase. None of these are ideal outcomes.

Firstly, while individual patient consent should be obtained when possible, it is not always practical to do so, especially retrospectively for data already collected for a different purpose [14]. Without consent and without an acceptable method for de-identification, data custodians are likely to become increasingly reluctant to disclose health data at all. Even when consent can be obtained, the disparity between consenters and non-consenters is significant. These two groups differ in demographic and socio-economic characteristics, resulting in biased data sets [15], [16], [17].

Secondly, if there is reduced incentive to de-identify health data when it is disclosed to serve important societal needs, more identifiable information will be disclosed instead [13]. It would be a greater risk to patient privacy if more identifiable information is disclosed when de-identified information would have satisfied the purpose.

Thirdly, if more identifiable data are disclosed for secondary purposes, there are real dangers from data breaches. The number of records affected by breaches is already quite high: the U.S. Department of Health and Human Services (HHS) has reported 252 breaches at health information custodians (e.g., clinics and hospitals) each involving more than 500 records from the end of September 2009 to the end of 2010 [18]. In all, the records of over 7.8 million patients have been exposed. If there are no requirements to de-identify data, society risks an avalanche of data breaches involving identifiable information requiring notification of the affected patients. A rising number of data breach notifications will erode the public's trust in data custodians [19], [20].

The argument that data is readily susceptible to re-identification is not new. In the 1990's, there was a well-publicized re-identification attack on a claims database containing information on 135,000 patients disseminated by the Group Insurance Commission [21]. In that attack, the discharge record for the then Governor of Massachusetts was re-identified using simple demographic information found in the Cambridge voter registration list which was purchased for $20. This was possible because certain fields in the two databases matched, namely: date of birth, 5-digit residential ZIP code, and gender. Since then, other examples of re-identification attacks have been reported on quite heavily by the media, including those of the web search queries of over a half-million America Online (AOL) clients [22] and the movie reviews of a half-million Netflix subscribers [23].

At first glance, it seems as if there are examples demonstrating a failure of de-identification. However, there has been no formal investigation to assess this evidence, and in particular, to contrast the re-identification attacks on health data with other types of data. Given the sensitivity of health information and potential implications for health policy (for example see [24]), it is critical to appraise the evidence in this domain.

We therefore performed a systematic review to: (a) characterize known re-identification attacks on health data and contrast that to re-identification attacks on other kinds of data, (b) compute the overall proportion of records that have been correctly re-identified in these attacks, and (c) assess whether these demonstrate a failure of current de-identification methods.

## Methods

We performed a systematic review of the relevant evidence demonstrating successful re-identification attacks on data sets that may have had some transformations applied to hide the individuals' identity. We examined articles from a wide array of communities reporting on such attacks, including statistics, computer science, and health informatics.

### Search Method

Articles in the statistical disclosure control literature, computer science literature, and medical informatics literature were searched by KEE and EJ using the general terms “anonymization”, “de-identification”, and “re-identification” indexed before the end of October 2010. Broad search terms were chosen to ensure that we did not miss any relevant publications. The searches were performed on PubMed, IEEE Xplore (the on-line library of the Institute of Electrical and Electronics Engineers) and the ACM Digital Library (the on-line library of the Association for Computing Machinery), and the records for all relevant English language articles were obtained for further consideration. The IEEE and ACM publish and index a significant amount of the computer science and medical informatics research work. The resulting set of articles was augmented with articles known to the authors, identified through targeted searches on Google Scholar (e.g., for specific authors), and articles identified through the reference lists of the included studies. Technical reports and presentations were also included.

### Inclusion/Exclusion Criteria

In total 1498 articles were identified from the databases and 24 from other sources. The article titles, keywords and abstracts were screened, where the primary inclusion criterion was that an article described a re-identification attack on an actual data set or a quantitative re-identification risk assessment. While we are mostly interested in the former, we included the latter during screening because it is often difficult to distinguish between the two types of articles from a title, keywords, and abstract.

To evaluate the accuracy of the screening, we performed an inter-rater reliability analysis with two independent raters. After the first rater completed his screening (KEE), a second rater not involved in the study in any way was recruited (KA). We went through the study objectives and screening criteria with the second rater to ensure consistency. For deciding how many articles needed to be rated we performed a power analysis for using the Kappa statistic [25] given an expected effect size of 0.8 at a power of 80% [26], [27]. We therefore required 18 articles to be screened by the second rater. We randomly selected 9 articles that were screened in by the first rater and 9 that were screened out by the first rater. The value of Kappa was found to be 0.85 (2-sided p<0.001).

Records that passed screening were obtained and assessed for eligibility through a full-text review. Articles were considered eligible if they went beyond a risk assessment and actually re-identified individuals. Studies which evaluated the risk of re-identification but did not attempt to re-identify any individuals were excluded (even if it was plausible in theory to re-identify individuals, if actual re-identification was not demonstrated then the article was excluded), for example, see [28], [29], [30], [31] for articles that were excluded. Furthermore, simulated attack studies, on artificial or real data, were excluded if they did not re-identify individuals.

We did not limit the selected articles to those that examined health data, but we did exclude studies examining the re-identification of genomic information. There is evidence that raw genomic information and summary statistics can distinguish individuals [32], [33], [34], and existing de-identification methods do not provide strong privacy guarantees [35]. Therefore, the assessment of re-identification risk from genomic information remains an active area of research [36].

The full-text of articles that made it through the two stage screening process were reviewed and abstracted. Two of the authors (KEE and EJ) characterized every article and where there were disagreements they were discussed and a consensus was reached for the final rating.

### Data Abstraction

The following six criteria were used to summarize each eligible study: (a) inclusion of health data in the attack, (b) the profession of the adversary, (c) country of re-identification, (d) the proportion or number of individuals re-identified, (e) whether the de-identification of the original data followed existing standards, and (f) whether the re-identification was verified. The first four criteria are descriptive, and characterize the nature and scope of successful re-identification attacks, whereas the latter two are quality indicators for the attack. These criteria were reviewed by a panel of five privacy experts, and were presented to a dozen privacy practitioners to solicit their feedback. While not comprehensive, these criteria were believed to provide a necessary foundation to understand and judge the nature of the re-identification attacks.

#### 3.1 Inclusion of health data

There tend to be sector-specific health privacy laws in many jurisdictions, arguably, resulting in health information being better protected than other types of information. Also, not all data sets are structurally the same. Each type of data set requires its own de-identification and re-identification methods. A re-identification attack on health information would therefore carry more weight in demonstrating the real-world risk of re-identification of health data.

#### 3.2 The profession of the adversary

Who is re-identifying data sets helps characterize the degree to which re-identification attacks are widespread. For example, if many different professions of adversaries are launching successful re-identification attacks and they vary in skill and resources, then this may indicate the ease with which re-identification attacks can occur.

#### 3.3 The country of re-identification

This refers to both the country of the adversary and the country where the individuals covered by the data come from. This characteristic is important because some countries make population databases readily available for free or for a modest fee. A good example of such publicly available population databases are state-level voter registration databases in the US [37]. There is also a thriving industry specializing in the creation and sale of databases containing personal information about the population, making a successful re-identification attack on a de-identified data set more likely [38].

#### 3.4 The percentage/number of individuals re-identified

The percentage (or number if no denominator is provided) of individuals re-identified is an indication of the severity of the re-identification attack. If a large percentage of records in a database were re-identified then it is a more severe attack than if a single individual has been re-identified, for example.

#### 3.5 The de-identification of the original data followed existing standards

If a data set that has not been de-identified in a defensible way is subsequently re-identified, then a successful re-identification attack on that data is not informative about how well the de-identification worked. Therefore, the method of de-identification is important to consider.

The US Health Insurance Portability and Accountability Act (HIPAA) Privacy Rule provides the most precise description of how to de-identify data among privacy laws in the US and Canada. In fact, the provisions of HIPAA have been applied in other jurisdictions. For example, health research organizations in Canada choose to use HIPAA standards to de-identify data sets [39], Canadian sites conducting research funded by US agencies need to comply with HIPAA [40], and international guidelines for the public disclosure of clinical trials data have relied on HIPAA definitions [41].

There are two de-identification standards specified in the HIPAA Privacy Rule: (a) the Safe Harbor standard, and (b) the statistical standard [3]. The former standard is quite precise in that it specifies 18 data elements that must be removed (e.g., patient names, full dates, and full ZIP code). These 18 elements are provided in Table 1. The latter standard requires that: (a) a statistical expert performs the de-identification, (b) the risk of re-identification is “very low”, and (c) the de-identification method is documented. Both standards ensure that the risk of re-identification is low, but not zero.

**Table 1:** The 18 elements in the HIPAA Privacy Rule Safe Harbor standard that must be removed or generalized for a data set to be considered de-identified (see 45 CFR 164.514(b)(2)(i)).

The following identifiers of the individual or of relatives, employers, or household members of the individual, are removed:
(A) Names;
(B) All geographic subdivisions smaller than a State, including street address, city, county, precinct, zip code, and their equivalent geocodes, except for the initial three digits of a zip code if, according to the current publicly available data from the Bureau of the Census: (1) The geographic unit formed by combining all zip codes with the same three initial digits contains more than 20,000 people; and (2) The initial three digits of a zip code for all such geographic units containing 20,000 or fewer people is changed to 000.
(C) All elements of dates (except year) for dates directly related to an individual, including birth date, admission date, discharge date, date of death; and all ages over 89 and all elements of dates (including year) indicative of such age, except that such ages and elements may be aggregated into a single category of age 90 or older;
(D) Telephone numbers;
(E) Fax numbers;
(F) Electronic mail addresses;
(G) Social security numbers;
(H) Medical record numbers;
(I) Health plan beneficiary numbers;
(J) Account numbers;
(K) Certificate/license numbers;
(L) Vehicle identifiers and serial numbers, including license plate numbers;
(M) Device identifiers and serial numbers;
(N) Web Universal Resource Locators (URLs);
(O) Internet Protocol (IP) address numbers;
(P) Biometric identifiers, including finger and voice prints;
(Q) Full face photographic images and any comparable images; and
(R) Any other unique identifying number, characteristic, or code.

While these two standards are not perfect, their application would provide some assurance that a generally accepted and broadly reviewed methodology was used to de-identify the data. If a standard was not used then it is not possible to know whether the de-identification applied on a data set provided meaningful protection against re-identification.

Therefore, the criterion we use to decide when a data set is defensibly de-identified is if it meets either of the two standards in the US HIPAA Privacy Rule. We will refer to this as “standards-based de-identification”.

#### 3.6 Re-identification has been verified

Once the adversary has re-identified a record, the adversary should verify that the re-identification is correct using additional information. Verification may be simple to do in a demonstration attack where the data custodian has the correct identities associated with the records and can verify each re-identified record. On the other hand, verification may require contacting the re-identified individual directly to confirm the facts (e.g., that the individual has the disease or condition that is indicated in the attacked database), or contacting the re-identified individual's work, school, co-workers, family, or neighbors. In some situations verification can be indirect. For example, if a re-identification attack reveals sensitive health information about a famous person and that person does not deny the sensitive information, then that may be taken as indirect verification.

Verification of re-identification attacks is important for three reasons. First, re-identification is probabilistic. Even if the probability of a correct re-identification is high, a re-identification attack is not successful unless some means have been used to verify the correctness of that re-identification. It is likely that an adversary would find multiple records that match the target individual and would choose one of these with equal probability. However, it is not possible to know with certainty if the chosen record is the correct one without verification.

Second, real data sets have quality problems. For example, a date of birth may be entered incorrectly into a database, or the digits in a ZIP code transposed. Such data errors may result in a potential re-identification being incorrect, even if all of the characteristics of the individual and the fields in the record match exactly. Only verification will indicate whether or not the re-identification was correct.

Third, background information that the adversary uses for re-identification may be old or cover a different time period than that contained in the attacked data set. Data aging or period mismatch may mean that seemingly correct matches are incorrect. In such cases, verification of the re-identified individuals is critical to ensure correctness.

### Mean Re-identification Rate

The main outcome from a re-identification attack is the re-identification rate: the proportion of records that were correctly re-identified. We used a chi-squared test to determine if the study proportions 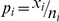 were homogeneous, where 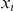 is the number of re-identified records, and 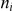 the database size, for study 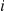. If they were homogeneous, we would treat the individual re-identification attacks as coming from the same general attack, thus estimating the overall proportion of re-identification attacks as simply 
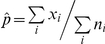
, known as the equal effects estimator. Otherwise, if they were not homogeneous, we would use the random-effects estimator proposed by Laird and Mosteller [42].

### Publication Bias

Re-identification attacks with a low re-identification rate are less likely to be published for two reasons: (a) it is less likely that an adversary will attempt to attack a data set with a low probability of correct re-identification, and (b) attacks that are performed and result in a low success rate are less likely to be published. We examine these two points below.

The overall probability of actual correct re-identification can be expressed as [43]:
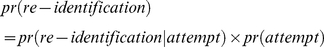
(1)This means that the overall probability of successful re-identification will depend on whether an adversary will attempt a re-identification to start off with. For the studies included in our review we know that 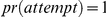. However, it is generally assumed that if the likelihood of successful re-identification is small then this would act as a deterrent for an adversary to attempt re-identification to start off with (i.e., if 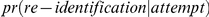 is low then 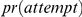 is also low) [43], [44]. By this reasoning, there will be fewer attacks attempted on data sets that have a very low likelihood of being re-identified by an adversary, such as those that have been de-identified using existing standards. The implication then is that we expect fewer studies with a low success rate to be published because they wouldn't be attempted.

If an adversary does attempt an attack, re-identification attacks with lower success rates are less likely to be published because, we would speculate, they are perceived by authors or journal and newspaper editors as less interesting.

On the other hand, an adversary may not wish to reveal a highly successful re-identification attack if the purpose of the attack is questionable. For example, one anecdote claimed that a banker used confidential information provided in loan applications to re-identify patients in a cancer registry with outstanding loans [45] - the details of such an attack would be unlikely to be published. Even if an attack was for demonstration or evaluation purposes, it may reveal that data were not sufficiently de-identified and the data custodian may not wish to reveal that fact.

If less successful attacks are less likely to be attempted or published, it would raise the overall mean proportion of records re-identified in our review. If more successful attacks are less likely to be published, it would reduce the overall mean proportion of records re-identified.

To examine these effects further, we computed the number of studies that would need to be performed and published to significantly change our mean proportion of records re-identified. This is similar to the computation of a failsafe N value to determine how many unpublished studies with null outcomes would be needed to change the significance of the results in a meta-analysis [46], [47]. In our analysis, instead of a single null outcome, we examined the sensitivity to a range of values for the proportion of records re-identified. We compared the number of studies required to change the mean proportion of records re-identified to a tolerance value based on the commonly used rule-of-thumb provided in the literature of 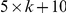, where 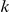 is the number of studies included in the analysis [46], [47].

We also evaluated publication bias using a funnel plot on the proportion of records re-identified [48]. This showed the proportion of records correctly re-identified against the standard error [49].

## Results

**Figure 1:**
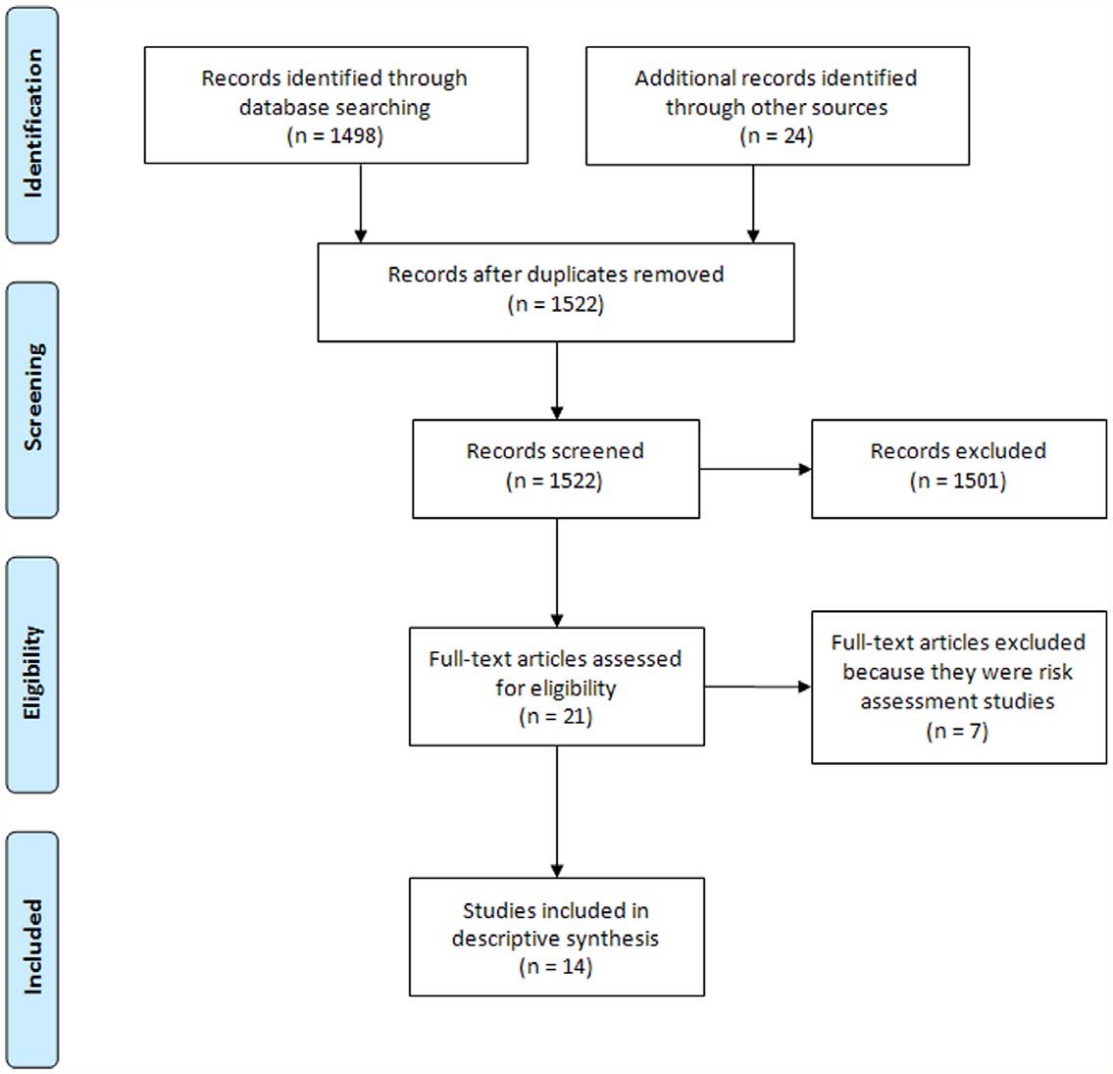
PRISMA diagram. PRISMA diagram summarizing the steps involved in the systematic review of the re-identification attack literature.

Figure 1 depicts a PRISMA diagram (Preferred Reporting Items for Systematic Reviews and Meta-Analyses) for this review [50], [51], and Checklist S1 contains the completed PRISMA checklist. We identified fourteen relevant studies as summarized in Table 2 according to our six criteria described in the “Methods” section.

**Table 2:** A summary of successful re-identification attacks on the evaluation criteria.

ID	Study	Pub Year ^§^	Health data included?	Profession of adversary	Number of individuals re-identified	Country of adversary	Proper de-identification of attacked data ?	Re-identification verified ?
**A**	[70]	2001	No	Researchers	29 of 273	Germany	“Factually anonymous”	Yes (records containing insurance numbers only)
**B**	[71]	2001	No	Researchers	75% of 11,000	USA	Direct identifiers removed	No
**C**	[67]	2002	Yes	Researcher	1 of 135,000	USA	Removal of names and addresses	Yes
	[56]	2003	No	Researchers	219 unique matches, 112 with 2 possibilities, 8 confirmed	UK	Yes	Verified matches, but not identities
**D**	[22]	2006	No	Journalist	1 of 657,000	USA	No	Yes (with individual)
**E**	[72]	2006	Yes	Researchers	79% of 550	USA	No	Verified (with original data set)
	[73]	2006	No	Researchers	Of 133 users, 60% of those who mention at least 8 movies	USA	Direct identifiers removed	No
**F**	[52]	2006	Yes	Expert Witness	18 of 20	USA	Only type of cancer, zip code and date of diagnosis included in request	Yes (verified by the Department of Health)
**G**	[74]	2007	No	Researchers	2,400 of 4.4 million	USA	Identifying information removed	Verified using original data
	[53]	2007	Yes	Broadcaster	1	Canada	Direct Identifiers removed & possibly other unknown de-id methods used	Yes
**H**	[23]	2008	No	Researchers	2 of 50	USA	Direct identifiers removed+maybe perturbation	No
**I**	[75]	2009	Yes	Researcher	1 of 3,510	Canada	Direct identifiers removed	Yes
**J**	[76]	2009	No	Researchers	30.8% of 150 pairs of nodes	USA	Identifying information removed	Verified using ground-truth mapping of the 2 networks
**K**	[57], [58] ^???^	2010	Yes	Researchers	2 of 15,000	USA	Yes - HIPAA Safe Harbor	Yes

(§This is the first year that the report or article appears. Some of the reports we cite have been updated at later dates. Some reports describe re-identification attacks that may have occurred in earlier years. 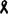 Since the appearance of the original results in 2010 a second article has been published more recently).

### Notable Observations

There are several notable observations from our review and the summary table that should be highlighted:

#### 1. Some studies did not report the attack methodology

Four re-identification attacks only reported the results briefly and had little description of the methodology used: one highly cited result was mentioned in passing as part of another study [21], an influential result had its methodology sealed as part of a court case [52], one was mentioned in an affidavit in a court case by a government official with no supporting information [53], and another example often cited by researchers and policy makers was described in a newspaper article with little description of the precise methodology followed [22]. The remaining 10 studies had more complete descriptions of their attack methodology.

#### 2 Few attacks involve health data

Six of the fourteen re-identification attacks involved health data. Even though they may influence the general perception of re-identification risk, successful re-identification attacks on other types of data (e.g., Internet search engine queries, movie ratings data, and relationships on social networks) do not necessarily translate into a real risk to health data, as opposed to successful re-identification attacks on health data.

#### 3. Most adversaries were researchers

Eleven of the fourteen successful re-identification attacks were performed by researchers to demonstrate that a risk exists or to evaluate if one exists, but not to exploit that risk (i.e., demonstration attacks). Only two of the fourteen attacks were conducted to inform a decision. These two re-identification attacks were on health data and both informed court judgments [52], [53]. The final attack was by journalists who wrote a newspaper article which resulted in the departure of the CTO at the data custodian, the dismissal of the individual responsible for the disclosure, and the data custodian not disclosing other data afterwards [54], [55]. Four out of the six health data attacks were performed by researchers.

#### 4. Most re-identification attacks were in the US

Ten of the attacks were performed by US-based investigators on data about or that included US citizens. This likely reflects a larger research community working on identifiability in the US and a greater availability of public and semi-public information for launching re-identification attacks. Four of the six re-identification attacks on health data were on US patient data, and two on Canadian patient data. The success of re-identification attacks will be jurisdiction-dependent because of variation in the availability of public and semi-public registers to use for matching. Successful attacks in the US will not necessarily succeed in other regions.

#### 5. Most re-identification attacks were verified

Eleven out of the fourteen studies, a significant proportion, did in fact verify their matches. All attacks on health data were verified. This is encouraging because it suggests thoroughness of work in this area.

#### 6. Most re-identified data was not de-identified according to existing standards

Only two of the studies were attacks on data de-identified in accordance with existing standards [56], [57], [58]. The remaining twelve attacks were committed against data that was left in varying degrees of an identifiable state, which only demonstrates that improperly de-identified data can be re-identified. Only one of the six re-identification attacks on health data was on a data set that was de-identified according to one of the existing standards, and it was found that the risk of re-identification was very low [57], [58].

The final point is best illustrated through several representative examples. First, recall the case of the re-identification of the Massachusetts governor. The information leveraged for re-identification was the date of birth, gender, and 5-digit residential ZIP code. These three features were not modified in any way prior to dissemination, which means that the claims database would not meet the Safe Harbor standard for de-identification. Second, AOL disclosed Internet search data on more than 675,000 of its users on a public website after replacing the users' names with persistent pseudonyms, but performed no de-identification of the search queries themselves. New York Times reporters were then able to determine the identity of a single individual in the data set from her search queries. However, the queries of the user in question included her town name and even her personal name. It is known that individuals often run search queries on their own names (i.e., vanity queries) and that their locations can be readily determined from the queries themselves [59], [60], [61], [62], [63], which makes it somewhat trivial to re-identify individuals from search queries. Third, the court case between the Southern Illinoisan newspaper and the public health department revolved around a cancer registry that included the patients' 5-digit ZIP code, which would not pass the Safe Harbor standard [52]. Finally, Netflix made a database of a sample of its subscribers' movie ratings publicly available for a data mining competition. The authors of the Netflix re-identification attack themselves stated that they believed very little perturbation or other form of de-identification was performed on the movie ratings data before they were disclosed [23]. In addition, there were dates included in the data set, which would make it fail the Safe Harbor standard.

Out of the fourteen attacks, in only two were the data de-identified according to current standards [56], [57], [58]. In these attacks, the risk of re-identification was found to be very low. In the first case, the authors matched sample records from the UK Census with records from the general household survey. The re-identification risk from the sample census records had been evaluated in detail by a team of statisticians, was known to be very low, and was documented, and therefore meets the definition of standards-based de-identification [43], [64]. The survey data could only be obtained under very strict confidentiality conditions. It is important to recognize that neither set of records actually communicated the individuals' identities. Rather the authors of the study verified their matches through the Office of National Statistics which was privy to the corresponding individuals' identities. It was not clear from this study what the exact proportion of records that could be re-identified might be, but the absolute number of matched records was small. The second case was commissioned by HHS to determine the re-identification risk of data de-identified using the HIPAA Safe Harbor standard. This study indicated that 0.013% of the records could be correctly re-identified, which was consistent with previous estimates of the actual risk of re-identification under Safe Harbor [37], [65], [66].

### Mean Re-identification Rate

Only 11 out of 14 studies clearly reported a denominator, allowing us to compute the proportion of records re-identified. A chi-squared test of homogeneity across the studies failed at an alpha level of 0.05, indicating heterogeneity. Therefore a simple combination of the proportions is not warranted. We instead used the random-effects estimator proposed by Laird and Mosteller [42].

We believe that the intent of re-identification varied among studies, in that some only wanted to prove that it could be done and were therefore satisfied with re-identifying a single record [22], [67], whereas others were attempting to re-identify as many records as possible in the database [52], [57], [58]. Random-effects models take such between-studies variation into account (as opposed to fixed-effects models), but could give more relative weight to attacks on small databases compared to fixed-effects models [68].

In the case of the random effects estimator we assume an infinite population of 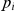
's, with mean 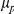 and variance 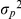. We weight the overall mean using the inverse of the within and between variance. That is, the weight for study 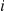 is 
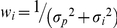
, where 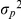 is the variance of the true 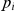
's , and 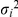 is the sampling variance for study 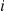. The overall mean is therefore estimated by weighted estimates of 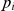, such that 
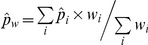
, with variance estimate 
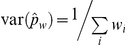
.

**Figure 2:**
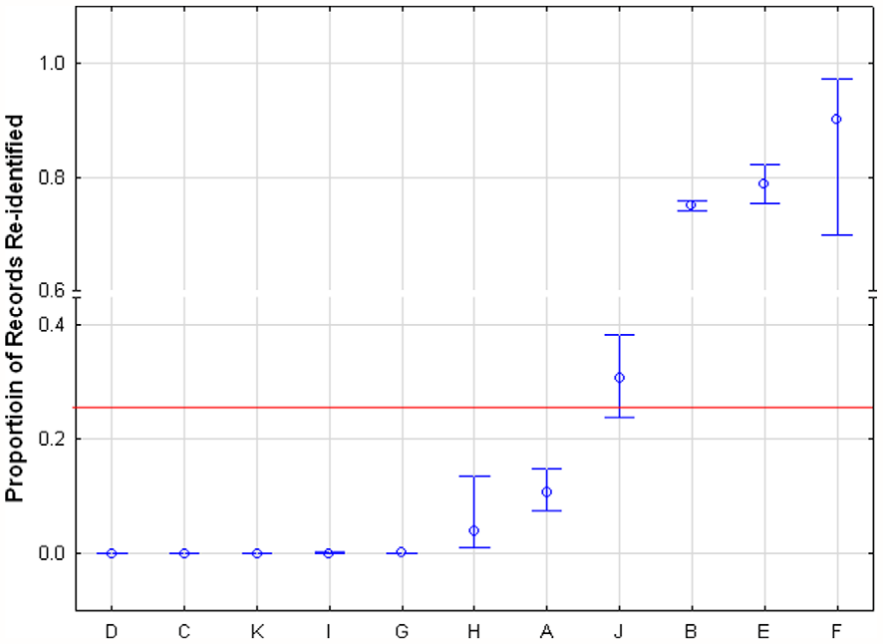
Caterpillar plot (all studies). Caterpillar plot of the individual mean and confidence intervals for all studies with overall mean proportion.

**Figure 3:**
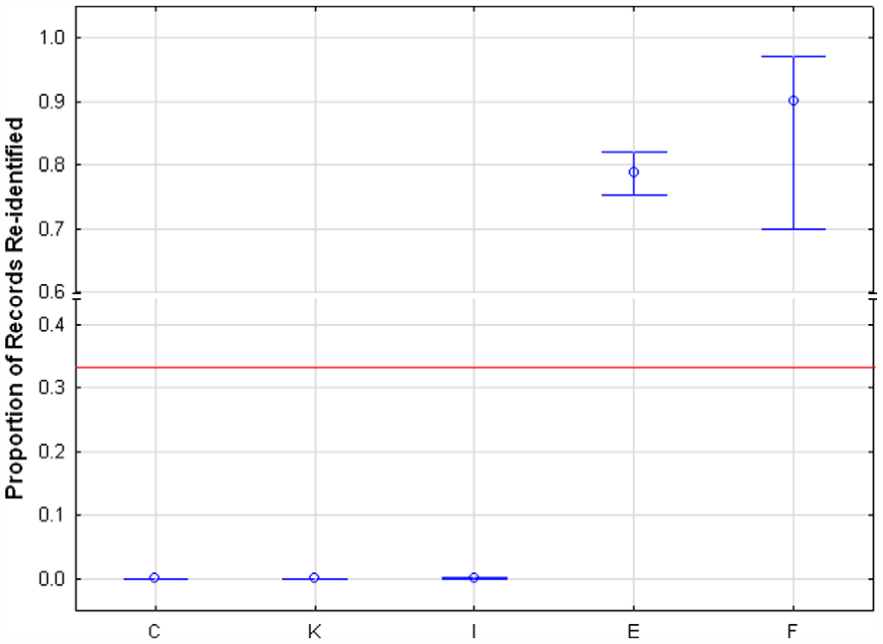
Caterpillar plot (health studies). Caterpillar plot of the individual mean and confidence intervals for health studies with overall mean proportion.

The confidence intervals for all studies that provided a denominator are shown in the caterpillar plot of Figure 2, and for only the health studies in the caterpillar plot of Figure 3. Caterpillar plots show the differences in the proportion of records re-identified among studies, and how they vary from (and affect) the mean. The overall mean proportion of records re-identified for all studies was 0.262 with 95% CI 0.046–0.478, and for re-identification attacks on health data only was 0.338 with 95% CI 0–0.744. Given such high re-identification rates, it is not surprising that there is a general belief that re-identification is easy. But also, it should be noted that the confidence intervals are quite wide, indicating considerable uncertainty.

If we remove the studies that had performed standards-based de-identification, then the overall mean proportion across the remainder (the ones not performing standards-based de-identification) was 0.288 (95% CI: 0.056–0.52) and for those on health data only it was 0.42 (95% CI: 0–0.9). The proportion of records that can be correctly re-identified when the data are not de-identified using standards-based methods is quite high.

On the other hand, the single study which was performed on health data that was de-identified using standards-based methods found that only 0.013% of the records could be re-identified. The proportion of records that can be correctly re-identified when the data is de-identified using standards-based methods is very low.

### Publication Bias

There was only one published re-identification attack on health data that was de-identified using current standards, and it had a low success rate. Possible explanations for the low publication rate of studies that have a low success rate are that: (a) there were fewer attacks attempted on data that has been de-identified using existing standards, and (b) attacks with a low success rate are less likely to be published.

Also twelve of fourteen studies were demonstration attacks performed by highly qualified experts in the field, which would mean that they would likely have higher success rates than those that weren't demonstration attacks. Although, as noted earlier, it is not necessary that all attacks with high success rates will be published, especially if they were not demonstration attacks since there would be less incentive to publicize them.

**Figure 4:**
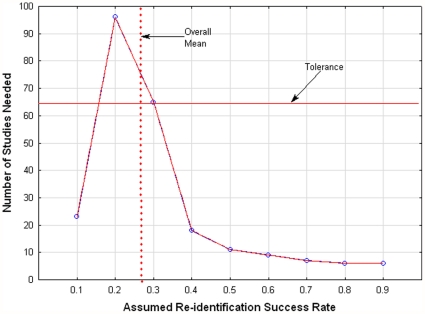
Senstivitiy (all studies). The number of new studies with success rates below/above the current mean that would need to be performed to significantly change the current mean for all studies.

**Figure 5:**
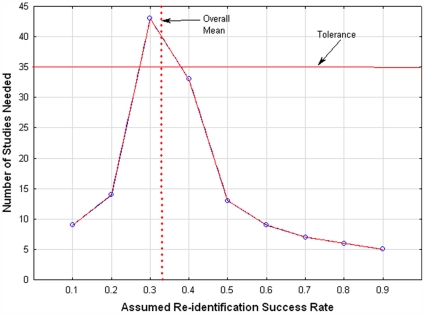
Sensitivity (health studies). The number of new studies with success rates below/above the current mean that would need to be performed to significantly change the current mean for health studies.

To examine this more systematically, we computed the number of unpublished studies with re-identification success rates below/above the current mean proportion of records re-identified (i.e., the 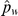 value) that would be needed to significantly decrease/increase that 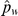 value. Initially, we assumed that studies with a re-identification success rate of 0.1 were done and not published. We can see in Figure 4 that 23 studies would have to exist such that the upper tail of the new 95% confidence interval would be below the current mean of 0.262. Similarly, 65 studies with a success rate of 0.3 would need to exist such that the lower tail of the new 95% confidence interval would be above the current mean of 0.262. A graph is plotted for different values of assumed success rate for all studies in Figure 4 and for health studies only in Figure 5. These graphs show as a horizontal line the tolerance value, which reflects the plausible number of unpublished studies (the “tolerance”). If the number of studies is below the tolerance value then there is cause for concern about the potential sensitivity of the results to unpublished studies. In general we can see that under most conditions the mean proportion value is sensitive to the existence of unpublished studies that show lower or higher re-identification success rates.

**Figure 6:**
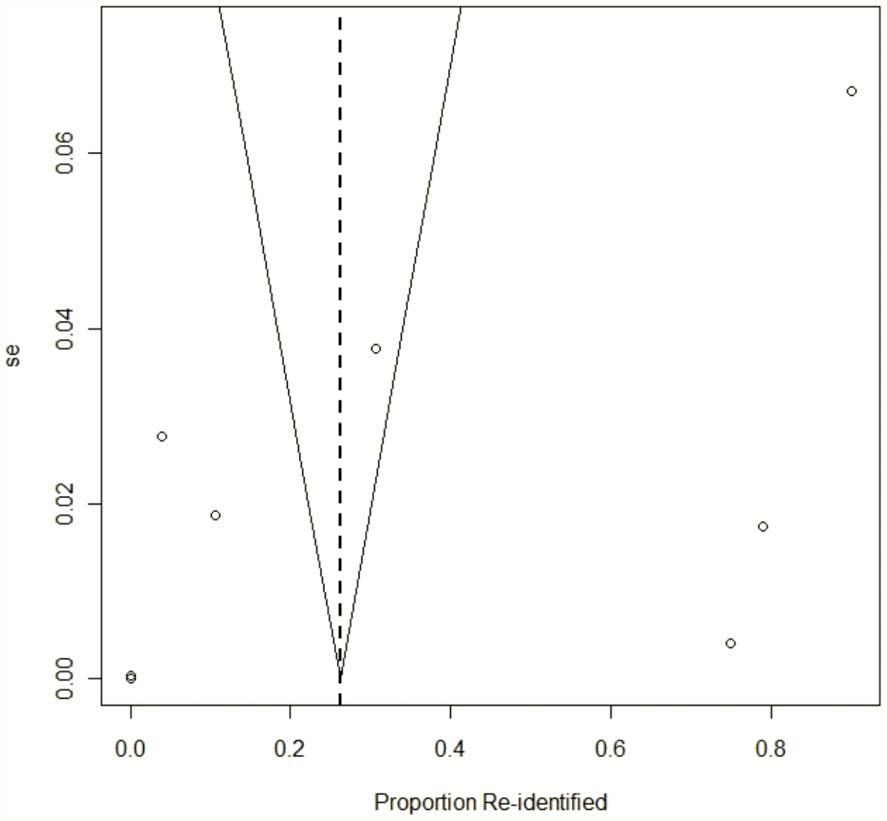
Funnel plot (all studies). Funnel plot showing the proportion of records re-identified in all studies against standard error. The points were slightly jittered to reveal overlap.

**Figure 7:**
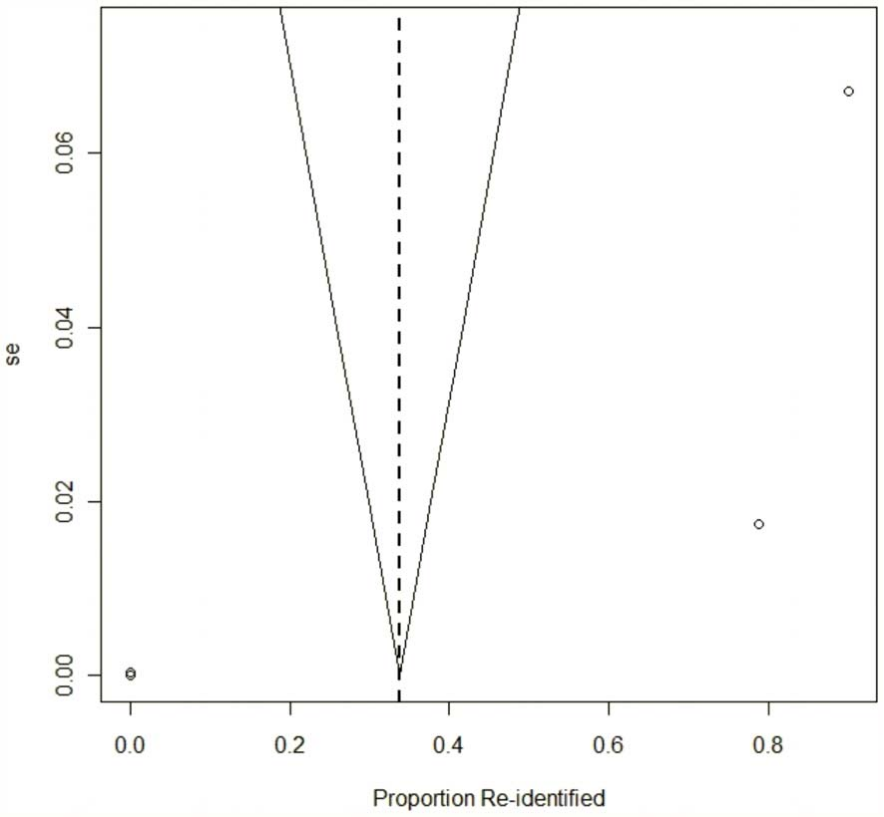
Funnel plot (health studies). Funnel plot showing the proportion of records re-identified in health studies against standard error. The points were slightly jittered to reveal overlap.

Figure 6 is a funnel plot for published re-identification attacks. This figure is consistent with considerable heterogeneity across studies. As expected, there was significant variation in the proportion of records that were re-identified for studies on small databases (those with higher standard errors). Studies on larger databases tended to have a small success rate (these are clustered around the origin). There were no studies on large databases with a high proportion of records re-identified. The same pattern is amplified for health data in Figure 7. This may be because it is difficult to re-identify many records in a large database (e.g., due to expense and time, and the technical challenges of doing so), or because large databases tend to be better de-identified and therefore have a low re-identification probability.

## Discussion

It is not surprising that policy makers believe that the success rate from re-identification attacks is high. The overall success rate for all re-identification attacks was approximately 26%, and 34% for health data. However, these results mask a more nuanced picture that makes it difficult to draw strong conclusions about the ease of re-identification.

The confidence interval around the above estimates was large, partially because many of the attacks were on small databases. Therefore, there is considerable uncertainty around these numbers.

We found only two studies where the original data was de-identified using current standards and for those the data was successfully re-identified. Only one of these attacks was on health data, and the percentage of records re-identified was 0.013%, which would be considered a very low success rate.

The number of unpublished studies that need to exist for the overall re-identification attack success rates to be shifted up or down was found to be plausible, meaning that the results are sensitive to unpublished attacks. Less successful attacks may not be published if they are perceived as not interesting. More successful attacks may not be published because they could potentially be embarrassing or cause difficulties to the adversaries and/or data custodians if exposed.

Finally, there was considerable heterogeneity among the studies. This makes it difficult to draw strong conclusions from the combined effect estimate of the proportion of records re-identified.

Future research in this area should focus on re-identification attacks on large databases that have been de-identified following existing standards, and success rates should be correlated with how well de-identification was performed. Metrics for measuring the extent of de-identification have been summarized elsewhere [69]. It is only then that we will have an evidence-based understanding of the extent to which de-identification protects against real attacks.

Meanwhile, the evidence suggests that it would be prudent for data custodians to continue to de-identify their data using current best practices. At the same time, due diligence should be applied: data custodians should complement such technical privacy protections with legal safeguards where appropriate, such as data sharing agreements which prohibit re-identification attempts and provide for accountability of one's actions.

## Supporting Information

Checklist S1 **PRISMA checklist indicating where in the paper the various items have been addressed.**
(DOC)
