# Correction: A Systematic Review of Re-Identification Attacks on Health Data

**DOI:** 10.1371/journal.pone.0126772

**Published:** 2015-04-16

**Authors:** Khaled El Emam, Elizabeth Jonker, Luk Arbuckle, Bradley Malin

The following information is missing from the Competing Interests section: KEE is the founder of Privacy Analytics Inc.

The complete, correct Competing Interests information is as follows: The authors have read the journal's policy and have the following conflicts: all co-authors perform consulting to federal and provincial governments and commercial entities in the US and Canada on de-identification. KEE is the founder of Privacy Analytics Inc, and KEE and BM sit on federal and provincial government advisory committees related to health information privacy. This does not alter the authors' adherence to all the PLOS ONE policies on sharing data and materials.

## References

[1] El Emam K, Jonker E, Arbuckle L, Malin B (2011) A Systematic Review of Re-Identification Attacks on Health Data. PLoS ONE 6(12): e28071 doi:10.1371/journal.pone.0028071 22164229 10.1371/journal.pone.0028071 PMC3229505

